# Bisphenol a Induces Autophagy Defects and AIF-Dependent Apoptosis via HO-1 and AMPK to Degenerate N2a Neurons

**DOI:** 10.3390/ijms222010948

**Published:** 2021-10-11

**Authors:** Ching-Tien Lee, Cheng-Fang Hsieh, Jiz-Yuh Wang

**Affiliations:** 1 Department of Nursing, Hsin-Sheng College of Medical Care and Management, Taoyuan 32544, Taiwan; chingtien1213@gmail.com; 2 Department of Neurology, Kaohsiung Medical University Hospital, Kaohsiung 80756, Taiwan; skywalker_hsieh@hotmail.com; 3 Division of Geriatrics and Gerontology, Department of Internal Medicine, Kaohsiung Medical University Hospital, Kaohsiung 80756, Taiwan; 4 Graduate Institute of Medicine, College of Medicine, Kaohsiung Medical University, Kaohsiung 80708, Taiwan; 5 Department of Medical Research, Kaohsiung Medical University Hospital, Kaohsiung 80756, Taiwan; 6 Neuroscience Research Center, Kaohsiung Medical University, Kaohsiung 80708, Taiwan

**Keywords:** Bisphenol A, heme oxygenase-1, AMPK, apoptosis, autophagy, neurodegeneration

## Abstract

Bisphenol A (BPA) is an environmental contaminant widely suspected to be a neurological toxicant. Epidemiological studies have demonstrated close links between BPA exposure, pathogenetic brain degeneration, and altered neurobehaviors, considering BPA a risk factor for cognitive dysfunction. However, the mechanisms of BPA resulting in neurodegeneration remain unclear. Herein, cultured N2a neurons were subjected to BPA treatment, and neurotoxicity was assessed using neuronal viability and differentiation assays. Signaling cascades related to cellular self-degradation were also evaluated. BPA decreased cell viability and axon outgrowth (e.g., by down-regulating MAP2 and GAP43), thus confirming its role as a neurotoxicant. BPA induced neurotoxicity by down-regulating Bcl-2 and initiating apoptosis and autophagy flux inhibition (featured by nuclear translocation of apoptosis-inducing factor (AIF), light chain 3B (LC3B) aggregation, and p62 accumulation). Both heme oxygenase (HO)-1 and AMP-activated protein kinase (AMPK) up-regulated/activated by BPA mediated the molecular signalings involved in apoptosis and autophagy. HO-1 inhibition or AIF silencing effectively reduced BPA-induced neuronal death. Although BPA elicited intracellular oxygen free radical production, ROS scavenger NAC exerted no effect against BPA insults. These results suggest that BPA induces N2a neurotoxicity characterized by AIF-dependent apoptosis and p62-related autophagy defects via HO-1 up-regulation and AMPK activation, thereby resulting in neuronal degeneration.

## 1. Introduction

Bisphenol A (BPA) is a common endocrine-disrupting chemical widely used in the manufacture of polycarbonate plastics and epoxy resins, and people inevitably come into contact with it in daily life [1,2,3,4]. Growing evidence suggests close correlations between BPA exposure, pathogenetic brain degeneration (e.g., Alzheimer’s disease (AD)), and altered neurobehaviors (e.g., cognitive decline) [5,6], which implies that BPA is an emerging risk factor for cognitive dysfunction. Low-dose BPA exposure inhibited 17β-estradiol-induced hippocampal synaptogenesis [7]. In rodents, exposure to BPA induced anxiolytic behaviors and cognitive deficits and caused persistent aberrations in spatial learning/memory [8,9]. BPA elicited AD-like neurotoxicity, exampled by substantially increased AD-associated pathological proteins in BPA-treated SH-SY5Y cells and adult mice [10,11]. Nevertheless, the mechanisms accounting for the neurotoxic BPA effects on brain development and AD-related neurodegeneration remain largely unknown.

Apoptosis and autophagy are two major modes of cellular self-degradation that play an important role in brain neurogenesis and neurodegeneration [12,13]. Apoptosis invariably contributes to cell death, but autophagy plays dual roles in cell survival and death depending on the cellular context [14]. Several studies describe the complex relationship of apoptosis and autophagy in various situations, including neurodegeneration [15], and propose that autophagy is a prerequisite for apoptosis, relieves apoptotic death, and, in combination with apoptosis, promotes cell death [16]. Current research on apoptosis and autophagy is focused on understanding the complex interplay between these two processes to discover novel strategies to prevent or treat neurodegeneration; however, thus far, little is known regarding the crosstalk of both in BPA-induced neurodegeneration.

Given the stress-inducible defense characteristics, heme oxygenase (HO)-1 induction is believed to provide adaptive protection against oxidative stress and neuroinflammation [17]. Considerable evidence supports the protective role of HO-1 in the central nervous system (CNS), which is ascribed to its anti-oxidative, anti-apoptotic, anti-inflammatory, and cytoprotective properties; however, some reports also highlight the harmful effects of up-regulated HO-1 in neuronal damage and brain degeneration [18,19,20,21]. HO-1 impaired synaptic plasticity to cause AD-like pathology and cognitive abnormality in mice [19]. Simvastatin-induced HO-1 enhanced apoptosis in Neuro-2a (N2a) neurons after glucose deprivation [20]. HO-1 inhibition offered neurotherapeutic effects in vitro and in transgenic AD mice [21]. Thus, the effect of HO-1 on neurodegeneration is ambiguous. The regulation of autophagy by HO-1 is another matter of concern. HO-1 prevented cardiac dysfunction by reducing apoptosis and enhancing autophagy in diabetic mice and promoted autophagy in podocytes to counteract against high glucose-induced apoptosis [22,23]. However, the effect of HO-1 on the modulation of apoptosis and autophagy in the context of BPA-induced neurotoxicity is unknown.

AMP-activated protein kinase (AMPK), a cellular energy sensor, is activated under stress conditions, such as ischemia, glucose deprivation, and oxidative stress [24,25]. To maintain energy homeostasis, AMPK phosphorylates its target raptor, thereby inhibiting the mammalian target of rapamycin to promote autophagy. Emerging evidence suggests the potential involvement of AMPK in controlling neural cell survival and death via regulating autophagy and apoptosis [25,26,27,28]. Pharmacological activation of AMPK causes cell cycle arrest and apoptosis in vitro and in vivo. However, AMPK activity also protects cells from metabolic stress and/or chemotherapy-induced apoptosis in certain conditions. Autophagy seems to play an important role in AMPK-dependent cytoprotection and cytotoxicity, and the final outcome may depend on the nature of the cytotoxic stimulus [29]. However, AMPK modulation of autophagy and apoptosis in BPA-induced neurotoxicity has not been clarified and, hence, requires further investigation.

We hypothesized that neuronal degeneration resulting from BPA cytotoxicity is due to the initiation of self-degradation responses involving HO-1 and AMPK. Herein, N2a cells possessing neurodifferentiation ability were used as a model, and our purpose is to explore the underlying mechanisms by which BPA negatively impacts N2a cell activities, including neuronal viability and differentiation. Signaling cascades related to apoptosis and autophagy were assessed after BPA treatment. We found that BPA induces N2a neurotoxicity characterized by p62 accumulation-related autophagy flux inhibition and apoptosis-inducing factor (AIF)-driven apoptosis through HO-1 up-regulation and AMPK activation. Thus, we proposed a toxicity mechanism showing that BPA directly induces neurotoxicity to account for the developmental defects and cognitive decline observed in brains chronically exposed to BPA.

## 2. Results

### 2.1. BPA Exerts Neurotoxic Effect to Impair the Development of N2a Neurons

**Figure 1:**
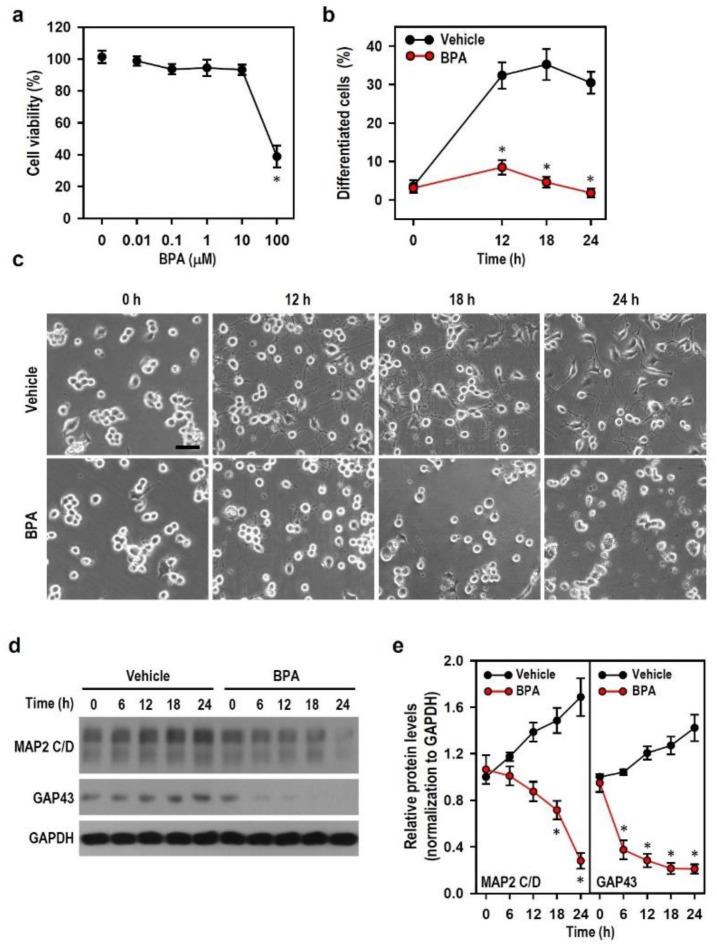
Bisphenol A (BPA) reduces cell viability and neuron-like differentiation in Neuro-2a (N2a) cells. (**a**,**b**) Cultures were treated with various BPA concentrations (0 to 100 μM) for 24 h and incubated with 100 μM BPA or vehicle for different time lengths (0 to 24 h). The untreated culture (0 μM BPA) was regarded as the control group. The cell viability and differentiation levels were separately determined by analyzing the MTT reduction capacity of the treated group relative to the control group (assigned a value of 100%) and by calculating the number of cells (% of total) bearing axon-like neurites longer than two cell-body in diameter. Each point represents the mean ± SEM (*n* = 4, triplicate). * *p* < 0.05 vs. control group (**a**) or vehicle group at identical time point (**b**). (**c**) Representative photos show cell morphology at specific time points after treating 100 μM BPA. Vehicle-treated culture served as a control. Neuron-like differentiation is indicated by the extent of axon outgrowth. Scale bar = 50 μm. (**d**,**e**) Cultures were treated with vehicle or 100 μM BPA for various time periods (0 to 24 h). Both levels of microtubule-associated protein 2 (MAP2) C/D and growth associated protein 43 (GAP43) were detected by Western blots. The line plots show the relative quantitation values normalized by GAPDH levels. The protein level at a time point of 0 h is assigned a value of 1. Data are represented as mean ± SEM (*n* = 4). * *p* < 0.05 vs. vehicle group at identical time point.

Given that BPA disturbs CNS neurons and chronically leads to neurodegeneration and brain developmental defects, we first explored whether BPA potentially impacts the developmental profiles of neurons. Cell viability and neuron-like differentiation were examined in N2a cell cultures exposed to BPA. We found that BPA was not toxic to cells until its concentration exceeded 10 μM. At the specific concentration of 100 μM, BPA significantly reduced cell viability and neuron-like differentiation compared with the untreated and vehicle-treated cultures (Figure 1a,b). Compared with vehicle-treated cultures, cultures treated with BPA revealed no further extension of a large number of short, sparse, and frequently broken neurites over time and a great amount of cell debris (Figure 1c). In comparison with vehicle-treated cells showing the time-dependent up-regulation of two neurodifferentiation markers, BPA-treated cells showed the marked down-regulation of growth associated protein 43 (GAP43) and microtubule-associated protein 2 (MAP2) levels at 6 and 18 h, respectively, post-treatment (Figure 1d,e). This finding implies that BPA interferes with axon extension in the early neurodifferentiation stage by negatively affecting the expression of differentiation-related factors. The immunofluorescence showed a weakening or disappearance of neuronal nuclear protein (NeuN, a marker of postmitotic neurons and differentiated cells) immunoreactivity in cultures treated with BPA instead of vehicle, indicating that damaged or dying neurons occur (Figure S1). Our results suggest that BPA impairs the viability and axon outgrowth of N2a cells, thus contributing to neuronal degeneration. Treatment with 100 μM BPA was used in subsequent experiments to further investigate the characteristics of BPA-induced N2a neuronal degeneration.

### 2.2. BPA Blocks Autophagy Flux and Induces Caspase-3-Independent Apoptosis in N2a Neurons

**Figure 2:**
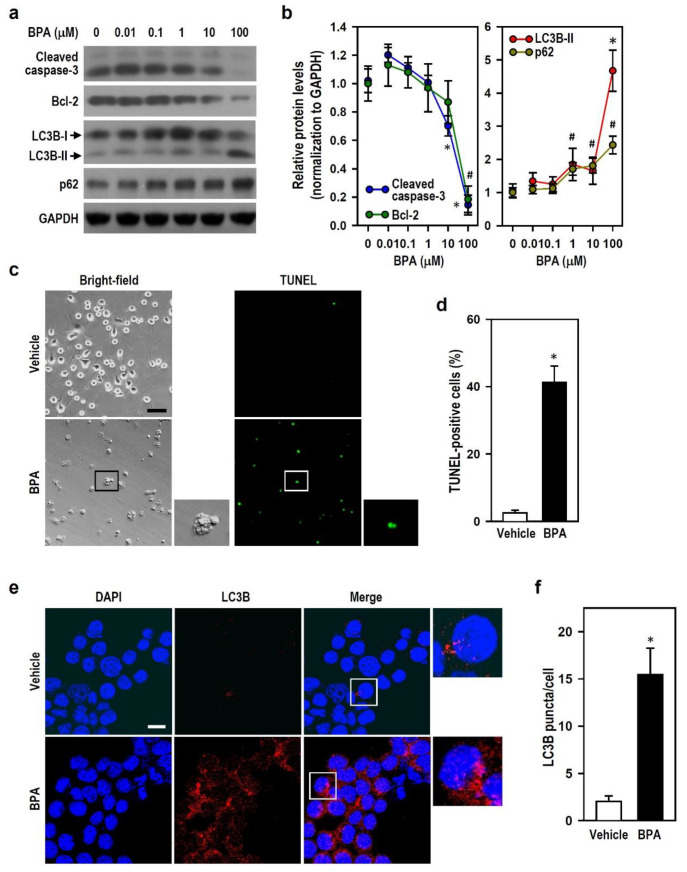
BPA interferes with autophagy and caspase-3-mediated apoptosis in N2a cells. (**a**,**b**) Cultures were treated with various BPA concentrations for 24 h. The levels of light chain 3B (LC3B), p62, cleaved caspase-3, and Bcl-2 were analyzed by Western blots. The line plots show the relative protein levels compared to untreated control group (0 μM BPA; assigned a value of 1). Each point represents the mean ± SEM (*n* = 4). * *p* < 0.05 vs. untreated group in cleaved caspase-3 or LC3B-II plot. # *p* < 0.05 vs. untreated group in Bcl-2 or p62 plot. (**c**,**d**) Terminal deoxynucleotidyl transferase-mediated dUTP nick end labelling (TUNEL) assay was performed 24 h after vehicle or 100 μM BPA treatment in cultures. Representative photos show the matching bright-field and TUNEL-FITC fluorescence (scale bar = 50 μm). For clarity, a selective area with higher magnification is displayed on the side. A bar plot shows the relative quantitation data expressed as the percentage of TUNEL-positive cells (green fluorescence). Values are represented as mean ± SEM (*n* = 3). * *p* < 0.05 vs. vehicle group. (**e**,**f**) Representative fluorescence images of LC3B staining are shown (scale bar = 20 μm). Cells grown on coverslips were harvested 24 h after vehicle or 100 μM BPA treatment. Immunostaining was executed using an antibody against LC3B (red), and the nucleus (blue) is marked by 4′,6-diamidino-2-phenylindole (DAPI). The side insert at higher magnification shows the pattern of LC3 puncta. A bar plot shows the relative quantitation results of LC3B immunoreactivity in cultures. Data are represented as mean ± SEM (*n* = 3). * *p* < 0.05 vs. vehicle group.

Cells that encounter environmental stress can initiate the machinery involved in programmed cell suicide to activate apoptosis and/or switch on the process of self-degradation, just like autophagy, to execute defensive protection. Thus, we examined whether apoptosis or autophagy occurs in N2a neurons exposed to BPA. Compared with BPA-untreated cultures (0 μM), cells treated with 100 μM BPA revealed decreased levels of cleaved caspase-3 (apoptotic effector) and anti-apoptotic Bcl-2 proteins (Figure 2a,b). In comparison with vehicle treatment, BPA treatment resulted in a marked increase in the percentage of terminal deoxynucleotidyl transferase-mediated dUTP nick end labelling (TUNEL)-positive N2a cells (Figure 2c,d). These data indicate that active caspase-3 is not involved in BPA-induced apoptosis. BPA caused evident increases in the level of light chain 3B (LC3B)-II, an autophagosome marker (Figure 2a,b). More LC3B puncta (red fluorescence) per cell were immunocytochemically detected in BPA-treated cultures compared with that in vehicle-treated cultures (Figure 2e,f). Because increases in LC3B-II can occur during induction of autophagy or inhibition of autophagic flux, we examined the cellular levels of p62 (an autophagy flux marker) to clarify BPA effect on autophagy. Autophagy induction often leads to p62 degradation [30]. Surprisingly, the p62 level in cultures treated with BPA increased markedly (Figure 2a,b), thereby indicating autophagy flux inhibition. These results suggest that BPA induces caspase-3-independent apoptosis and increases autophagosome content; however, the autophagic flux is blocked.

### 2.3. BPA Inhibits the Generation of Active Caspase-3, and BPA-Induced Autophagy Flux Inhibition Involves the Increases in LC3B-II and p62 Production

**Figure 3:**
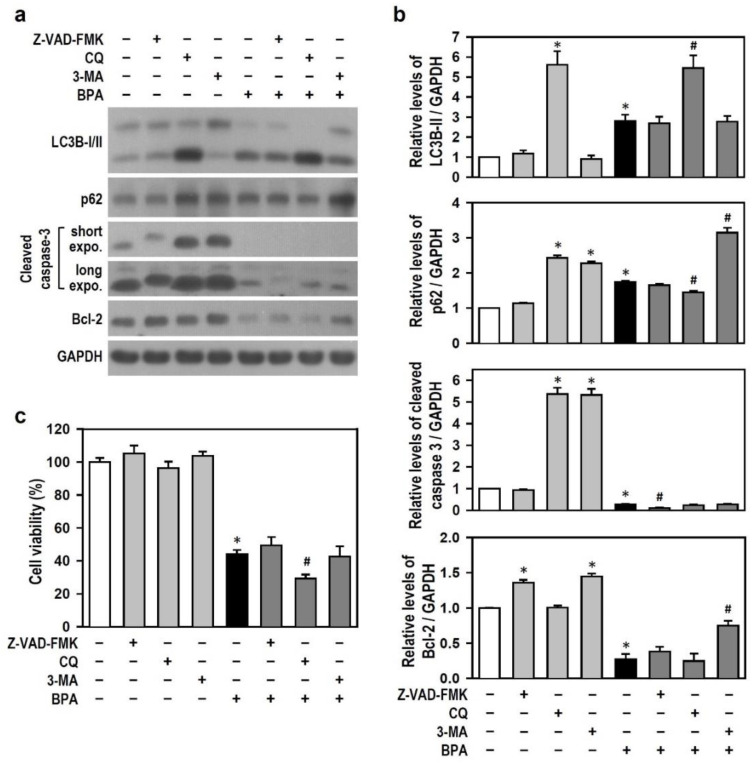
BPA induces autophagy flux inhibition and caspase-independent apoptosis in N2a cells. (**a**,**b**) Cultures were treated with pharmacological inhibitors, i.e., 40 μM Z-VAD-FMK, 30 μM chloroquine (CQ), and 2 mM 3-methyladenine (3-MA), for 30 min and then exposed or not to 100 μM BPA for 24 h. The untreated cultures served as a control. The levels of LC3B, p62, cleaved caspase-3, and Bcl-2 were analyzed by Western blots. The bar plots show the quantitative data (normalized by GAPDH) for each protein relative to the control group (assigned a value of 1). Data are represented as mean ± SEM (*n* = 4). * *p* < 0.05 vs. control group; # *p* < 0.05 vs. BPA alone. (**c**) Similar manipulation was performed as (**a**), and the cultures were harvested to determine cell viability. The untreated control group is assigned a survival rate of 100%. Data are represented as mean ± SEM (*n* = 4, triplicate). * *p* < 0.05 vs. control group; # *p* < 0.05 vs. BPA alone.

In order to confirm that BPA blocks caspase-3-related apoptosis and explore the connection between apoptosis and autophagy, we challenged BPA-treated N2a cells with chloroquine (CQ, an autophagic flux inhibitor), 3-methyladenine (3-MA, an autophagy inhibitor), and Z-VAD-FMK (a pan-caspase inhibitor) and then checked active caspase-3 levels. We observed that Z-VAD-FMK has no effect on LC3B-II and p62 but reduces cleaved caspase-3 levels in the presence of BPA. Treatment with CQ or 3-MA alone increased the cleaved caspase-3 level; however, this effect was blocked by BPA (Figure 3a,b). This implies that, regardless of whether or not autophagy is inhibited, BPA reduces the generation of active caspase-3, negatively impacting caspase-dependent apoptosis.

Additionally, to verify the inhibitory effect of BPA on autophagic flux, we examined the level of autophagy-related marker proteins. CQ alone inhibited the turnover of LC3B-II and p62, indicating a blockade of autophagic flux. The accumulation of LC3B-II and p62 caused by BPA was comparable with the effects of CQ (Figure 3a,b), suggesting that BPA mimics the CQ effect on autophagy flux inhibition. None of the inhibitors demonstrated a rescue effect on BPA-induced cell death, although 3-MA elevated Bcl-2 level (Figure 3a–c). CQ enhanced the effect of BPA in decreasing cell viability (Figure 3c), implying that the enhanced autophagic flux inhibition aggravates BPA insults.

Thus, the data suggest that BPA-induced apoptosis in N2a neurons is independent of caspase-3 and confirm that the elevated levels of LC3B-II and p62 caused by BPA are associated with autophagy flux inhibition.

### 2.4. BPA Shuts Down Caspase-3-Driven Apoptosis and Activates AIF-Related Apoptosis in N2a Neurons

**Figure 4:**
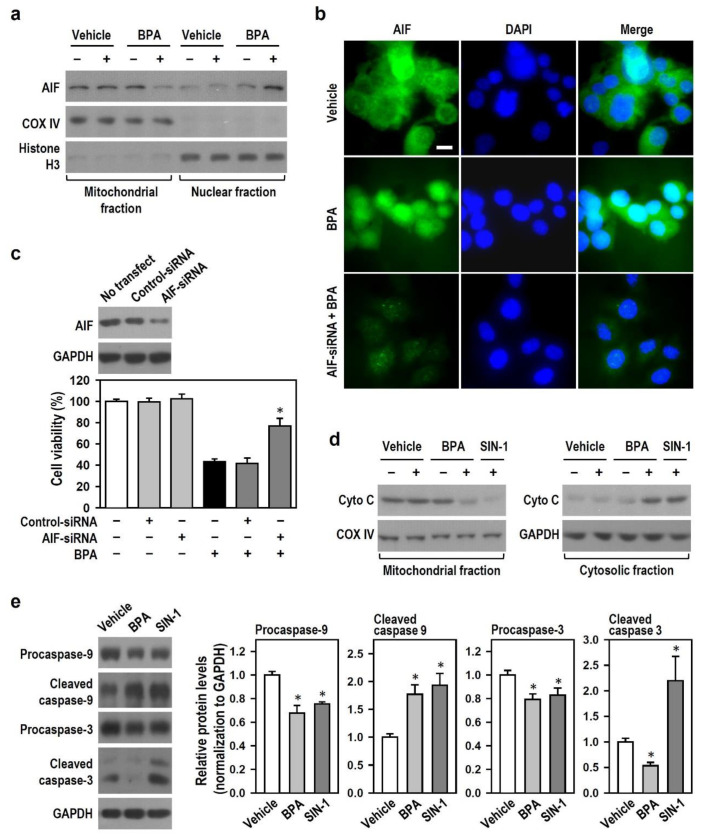
BPA impairs the cytochrome c/caspase-9/caspase-3 apoptotic axis and elicits a nuclear translocation of mitochondrial apoptosis-inducing factor (AIF) in N2a cells. (**a**) Cultures treated with vehicle or 100 μM BPA for 12 h were harvested and subjected to subcellular fractionation. AIF level was detected by Western blots. Cytochrome c oxidase (COX) IV and histone H3 were the markers of mitochondrial and nuclear subcellular fractions, respectively. (**b**) Representative fluorescence images of AIF immunoreactivity (green) are shown (scale bar = 10 μm). Cells grown on coverslips were harvested 12 h after vehicle or 100 μM BPA treatment. DAPI staining (Nucleus, dark blue) allows identification of the nuclear translocation of AIF (Merge, light blue). (**c**) Transient transfection of small interfering RNA (siRNA) was used to silence AIF protein formation. Cultures were untreated or treated with control- or AIF-siRNA (50 nM) as indicated, and the silencing efficacy was evaluated by Western blots. Additionally, the siRNA-transfected cultures were exposed or not to BPA for 24 h. Cell viability was assessed by MTT reduction assay. The bar plot shows the survival percent compared to the untreated control group (assigned a value of 100%). Values are represented as mean ± SEM (*n* = 4, triplicate). * *p* < 0.05 vs. BPA alone. (**d**) Similar manipulation was conducted as (**a**), and cytochrome c level was detected by Western blots. COX IV and GAPDH were mitochondrial and cytoplasmic markers, respectively. 3-morpholinosyndomine (SIN-1) at 1 mM was a positive control to induce apoptosis. (**e**) Cultures exposing to vehicle or the indicated agents for 24 h were harvested and subjected to Western blots to detect procaspase-9, cleaved caspase-9, procaspase-3, and cleaved caspase-3 levels. The bar plots show the relative quantitation data of these proteins. Values are represented as mean ± SEM (*n* = 4). * *p* < 0.05 vs. vehicle group.

Given that the above findings show that BPA down-regulates active caspase-3 and may induce caspase-independent apoptosis, we sought to explore the signaling cascades of caspase-dependent and -independent apoptosis. We examined whether AIF (a caspase-independent death effector) is involved and if the cytochrome c/caspase-9/caspase-3 axis is impaired in BPA-induced N2a neuronal death. Compared with the vehicle groups, groups treated with BPA for 12 h showed decreased AIF level in mitochondrial fraction and increased AIF level in nuclear fraction (Figure 4a). Immunofluorescence revealed the same results. Following BPA treatment, strong AIF-positive fluorescence (green) was observed inside the nucleus; in the vehicle group, AIF immunoreactivity was mainly located outside the nucleus (Figure 4b). AIF silencing by RNA interference (RNAi) erased the nuclear translocation of AIF and obviously attenuated BPA effect on cell survival decline (Figure 4b,c), implying that AIF plays a key role in BPA-induced N2a cell death. BPA treatment for 12 h decreased mitochondrial cytochrome c level, which coincided with an increase in cytosolic cytochrome c level (Figure 4d). 3-morpholinosyndomine (SIN-1) served as a positive control to recognize the induction of caspase-dependent apoptosis. Compared with the vehicle group, BPA elicited the cleavage of procaspase-9 and 3; however, the increased level was observed in cleaved caspase-9 but not in cleaved caspase-3, and even BPA lowered cleaved caspase-3 generation to a level below the baseline (Figure 4e). The above results suggest that AIF mediates the BPA effect on N2a neuronal apoptosis and that BPA switches off cytochrome c/caspase-9/caspase-3-driven apoptosis via the down-regulation of active caspase-3.

### 2.5. BPA-Induced HO-1 Mediates N2a Neurotoxicity via AMPK Activation, Autophagy Initiation, AIF-Dependent Apoptosis and Bcl-2 Down-Regulation

**Figure 5:**
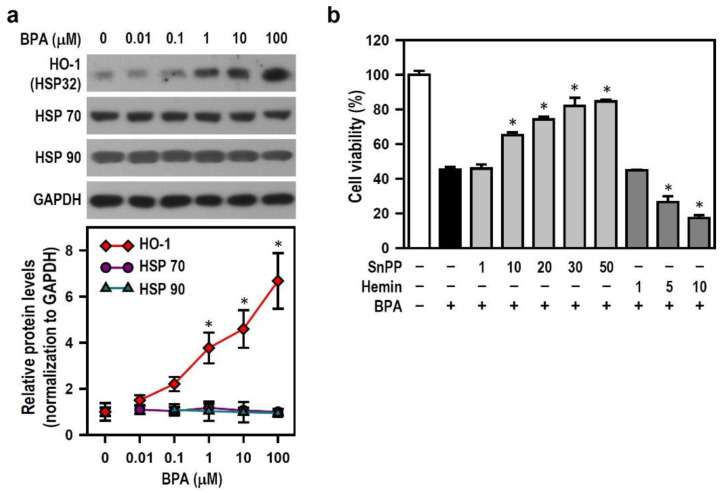
BPA selectively induces the production of heme oxygenase (HO)-1 but not HSP70 and HSP90, leading to the N2a cell survival decline. (**a**) Cultures were treated with different BPA concentrations for 24 h, and untreated culture (0 μM BPA) was regarded as the control group. HO-1, HSP70 and HSP90 levels were detected using Western blots. A line plot shows the relative quantitation data of these HSPs. Each point represents the mean ± SEM (*n* = 4). * *p* < 0.05 vs. control group. (**b**) Cultures were exposed to various concentrations (μM) of HO-1-related pharmacological agents, hemin chloride (Hemin), or tin protoporphyrin IX (SnPP), for 30 min before being treated with 100 μM BPA for 24 h. Cell viability was assessed by MTT reduction assay. A bar plot shows the survival percent relative to the untreated control group (assigned a value of 100%). Data are represented as mean ± SEM (*n* = 4, triplicate). * *p* < 0.05 vs. BPA alone.

**Figure 6:**
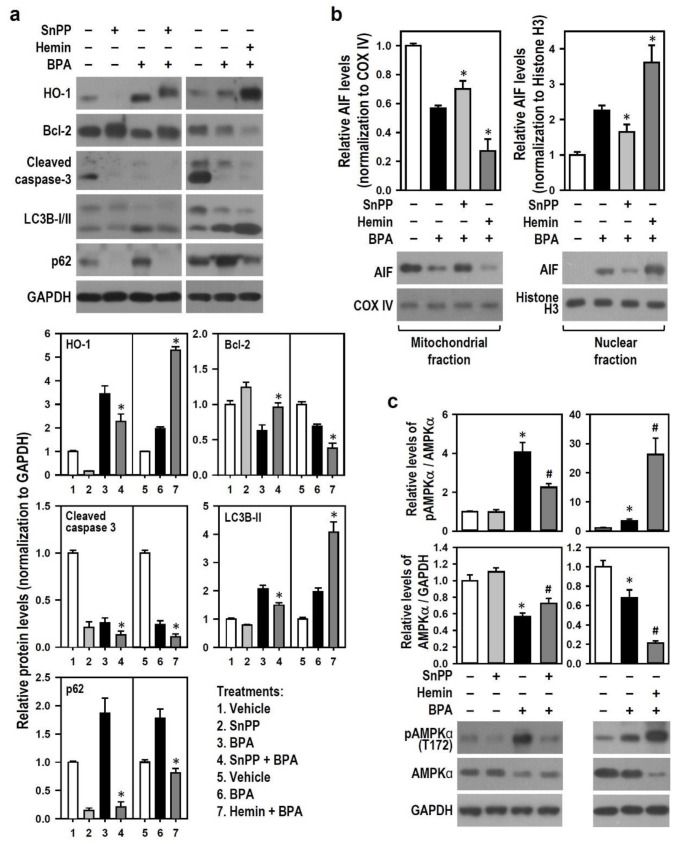
HO-1 mediates BPA effects on autophagy activation, nuclear translocation of AIF, and AMP-activated protein kinase (AMPK)α phosphorylation, as well as the down-regulation of cleaved caspase-3, Bcl-2, and AMPKα. (**a**) Cultures were exposed to 30 μM SnPP or 10 μM hemin for 30 min before being treated with or without 100 μM BPA for 24 h. HO-1, Bcl-2, cleaved caspase-3, LC3B, and p62 levels were detected by Western blots. The bar plots show the relative quantitation data of these proteins. Values are represented as mean ± SEM (*n* = 4). * *p* < 0.05 vs. BPA alone. (**b**) Cultures were pretreated with 30 μM SnPP or 10 μM hemin for 30 min and continuously incubated with 100 μM BPA for 12 h. Cultures were subjected to subcellular fractionation after harvest. AIF level was detected by Western blots. COX IV and histone H3 were markers standing separately for mitochondrial and nuclear fractions. The bar plots show the quantitative results relative to the untreated control group (assigned a value of 1). Data are represented as mean ± SEM (*n* = 4). * *p* < 0.05 vs. BPA alone. (**c**) Similar manipulation was conducted as (**a**), and pAMPKα and AMPKα levels were detected by Western blots. The bar plots show the quantitative values of pAMPKα (normalized by AMPKα) and AMPKα (normalized by GAPDH) relative to the untreated control group (assigned a value of 1). Data are represented as mean ± SEM (*n* = 4). * *p* < 0.05 vs. control group; # *p* < 0.05 vs. BPA alone.

Environmental toxins have been treated as stressors that elicit dramatic changes in neuronal cell activity. The stress response underlying cellular adaptation to adverse conditions is typically mediated by stress proteins. We examined the role of heat shock proteins (HSPs, including HO-1, HSP70, and HSP90) in BPA toxicity because these stress-inducible proteins have been extensively studied in experimental neurodegenerative models [31]. BPA concentration-dependently caused gradual increases in the level of HO-1, but not in those of HSP70 and HSP90 (Figure 5a). For further investigation, we used hemin chloride (hemin, a HO-1 inducer) and tin protoporphyrin IX (SnPP, a HO-1 inhibitor) in subsequent experiments. SnPP effectively relieved BPA insults, whereas hemin markedly enhanced BPA-induced declines in cell viability (Figure 5b); moreover, SnPP obviously reduced the HO-1 induction levels evoked by BPA, whereas hemin provided more HO-1 to enhance BPA-induced HO-1 production (Figure 6a). These data indicate that BPA-induced HO-1 mediates N2a cell death, and the HO-1 level is negatively correlated with neuronal survival.

Next, we examined whether HO-1 is involved in the BPA effect on N2a cell apoptosis and autophagy. Compared with BPA treatment alone, SnPP obviously increased Bcl-2 generation and decreased the levels of cleaved caspase-3, LC3B-II, and p62 in the presence of BPA. Hemin markedly strengthened the effects of BPA in decreasing Bcl-2 and cleaved caspase-3 levels and increasing LC3B-II level; additionally, hemin evidently inhibited BPA-induced increase in p62 level (Figure 6a). Furthermore, hemin significantly enhanced the BPA-induced translocation of AIF from mitochondria to nuclei, whereas SnPP evidently inhibited the AIF translocation caused by BPA (Figure 6b). Because AMPK can regulate neurodegeneration-related apoptosis and autophagy [27,28,32] and interact with HSPs in response to cellular stresses [33,34], we examined AMPK activity in BPA-treated N2a neurons. Compared with no treatment, BPA treatment evidently increased AMPKα phosphorylation level, indicating that AMPK activation is related to BPA toxicity in N2a neurons. Additionally, compared with BPA treatment alone, SnPP obviously reduced the AMPKα phosphorylation level induced by BPA, whereas hemin markedly enhanced BPA-induced AMPKα phosphorylation (Figure 6c), implying that HO-1 promotes AMPK activation.

Taken together, our results suggest that HO-1 selectively induced by BPA plays a key role in mediating BPA toxicity in N2a neurons via AIF-driven apoptosis, increased autophagic flux, AMPK activation, and Bcl-2 down-regulation.

### 2.6. BPA-Activated AMPK Reduces HO-1 Induction and Autophagy Initiation

**Figure 7:**
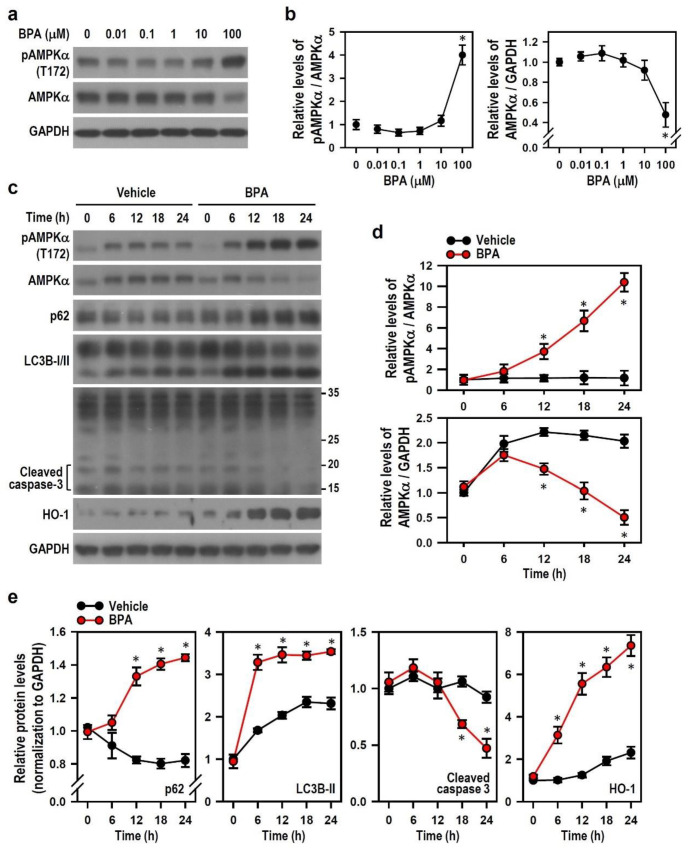
BPA enables activation of AMPK and time-dependently causes an AMPKα phosphorylation, accompanied by up-regulation of LC3B, p62, and HO-1 and down-regulation of cleaved caspase-3. (**a**,**b**) Cultures were treated with various BPA concentrations for 24 h. The levels of pAMPKα and AMPKα were detected by Western blots. The line plots show the quantitative data of pAMPKα (normalized by AMPKα) and AMPKα (normalized by GAPDH) relative to the untreated control group (0 μM BPA; assigned a value of 1). Each point represents the mean ± SEM (*n* = 4). * *p* < 0.05 vs. control group. (**c**–**e**) Cultures were treated with vehicle or 100 μM BPA for various time periods as indicated. The levels of pAMPKα, AMPKα, p62, LC3B, caspase-3, and HO-1 were detected by Western blots. The line plots show the relative quantitation values after normalization. The protein level at a time point of 0 h is assigned a value of 1. Data are represented as mean ± SEM (*n* = 4). * *p* < 0.05 vs. vehicle group at identical time point.

Given our finding that BPA induces AMPK activation, we further explored changes in AMPK activity at various BPA concentrations in N2a neurons. Increasing the BPA concentrations for 24-h treatment did not elevate AMPKα phosphorylation level markedly until 100 μM; a low AMPKα protein level was also clearly detected (Figure 7a,b). Short-term treatment with BPA caused an immediate increase in AMPKα phosphorylation within 5 min, followed by a slow dephosphorylation of the kinase over the next 1 h, suggesting that BPA also induces acute AMPK activation (Figure S2). Compared with the vehicle, BPA treatment for 24 h elicited increases in AMPKα phosphorylation over time and gradually decreased the AMPKα protein levels correspondingly (Figure 7c,d), implying that BPA activates and down-regulates AMPK over time. We further found that BPA treatment evokes progressive increases in the levels of p62, LC3B-II, and HO-1 and a gradual reduction in the level of cleaved caspase-3 (Figure 7c,e). Interestingly, the time-course changes of these proteins were parallel to changes in AMPK activation, which means BPA-activated AMPK may modulate protein formation levels. Thus, we applied 5-aminoimidazole-4-carboxamide riboside (AICAR, an AMPK activator) and dorsomorphin (an AMPK inhibitor) to examine whether AMPK plays a role in BPA-induced N2a cell death.

**Figure 8:**
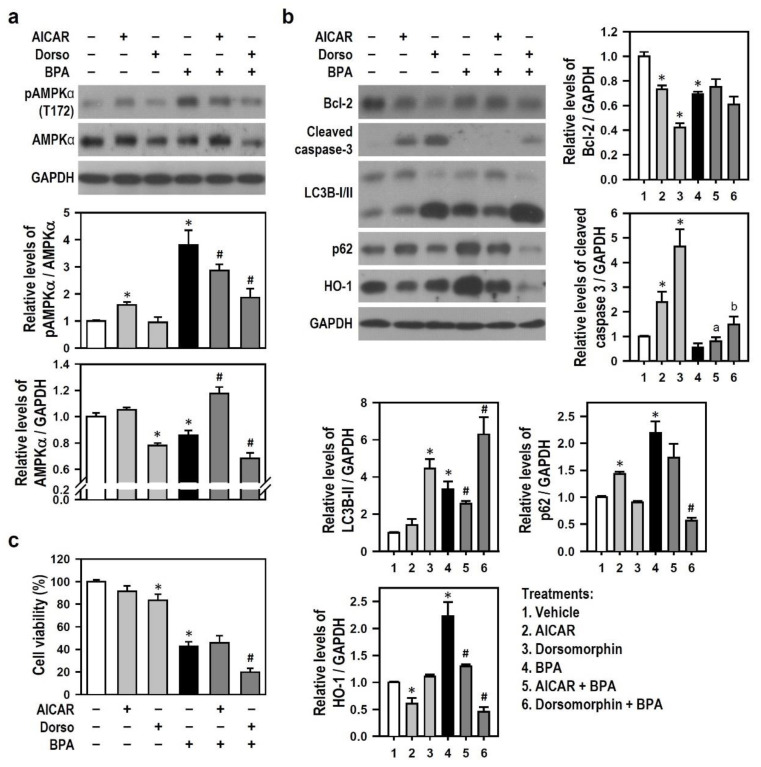
AMPK activated by BPA is related to the levels of p62 and LC3B; AICAR lessens BPA-induced formation of LC3B and HO-1, while dorsomorphin reduces BPA-induced p62 accumulation and aggravates N2a cell death caused by BPA. (**a**) Cultures were exposed to AMPK-related pharmacological agents, i.e., 2 mM 5-aminoimidazole-4-carboxamide riboside (AICAR) or 10 μM dorsomorphin (Dorso), for 30 min before being treated with or without 100 μM BPA for 24 h. The levels of pAMPKα and AMPKα were detected by Western blots. The bar plots show the quantitative data of pAMPKα (normalized by AMPKα) and AMPKα (normalized by GAPDH) relative to the untreated control group (assigned a value of 1). Data are represented as mean ± SEM (*n* = 4). * *p* < 0.05 vs. control group; # *p* < 0.05 vs. BPA alone. (**b**) Similar manipulation was performed as (**a**), and the levels of Bcl-2, cleaved caspase-3, LC3B, p62, and HO-1 were detected by Western blots. The bar plots show the quantitative data of these proteins (normalized by GAPDH) relative to the untreated control group (assigned a value of 1). Values are represented as mean ± SEM (*n* = 4). * *p* < 0.05 vs. control group; # *p* < 0.05 vs. BPA alone; a *p* < 0.05 vs. AICAR alone; b *p* < 0.05 vs. dorsomorphin alone. (**c**) Similar manipulation was performed as (**a**), and cultures were subjected to MTT reduction assay to assess cell viability. A bar plot shows the survival percent relative to untreated control group (assigned a value of 100%). Data are represented as mean ± SEM (*n* = 4, triplicate). * *p* < 0.05 vs. control group; # *p* < 0.05 vs. BPA alone.

We observed that AICAR alone stimulates AMPKα phosphorylation, whereas dorsomorphin obviously reduces AMPKα protein level. Compared with BPA treatment alone, dorsomorphin was superior to AICAR in markedly reducing BPA-induced AMPKα phosphorylation; further, AICAR nearly reversed BPA-induced AMPKα protein decline to the basal level, whereas dorsomorphin enhanced the AMPKα protein degradation caused by BPA (Figure 8a), meaning that AMPK activation by BPA stabilizes AMPKα protein level. As shown in Figure 8b, AICAR or dorsomorphin treatment alone reduced the Bcl-2 generation but did not alter the Bcl-2 down-regulation caused by BPA, indicating that BPA alters AMPK activity to down-regulate Bcl-2 via an effect similar to AICAR or dorsomorphin. Compared with the untreated control, AICAR alone increased the levels of p62 and cleaved caspase-3 but decreased HO-1 level; dorsomorphin alone elevated the levels of LC3B-II and cleaved caspase-3. These results indicate that AMPK affects HO-1 production and participates in the modulation of autophagy and apoptosis. Compared with BPA treatment alone, AICAR clearly decreased BPA-induced production levels of LC3B-II and HO-1, whereas dorsomorphin evidently enhanced the BPA-induced increase in LC3B-II level and reduced the p62 formation induced by BPA. These data indicate that BPA-activated AMPK exerts an antagonistic effect against BPA-induced HO-1 production and subsequent autophagy initiation. Although AICAR showed no effect against BPA insults, dorsomorphin notably increased the BPA effect on cell death, suggesting that inhibition of BPA-activated AMPK enhances BPA-induced N2a neurotoxicity (Figure 8c).

The above results suggest that activated AMPK causes p62 accumulation and affords partial protection by incompletely repressing the bulks of HO-1 and LC3B-II, thus leading to autophagy flux inhibition in N2a neurons exposed to BPA. Thus, blockade of AMPK activity aggravates BPA-induced N2a neuronal death via autophagic or apoptotic death.

### 2.7. Reactive Oxygen Species (ROS) Induced by BPA Involve AMPK Activation and HO-1 Induction but Does Not Mediate Apoptosis and Autophagy Flux Inhibition

**Figure 9:**
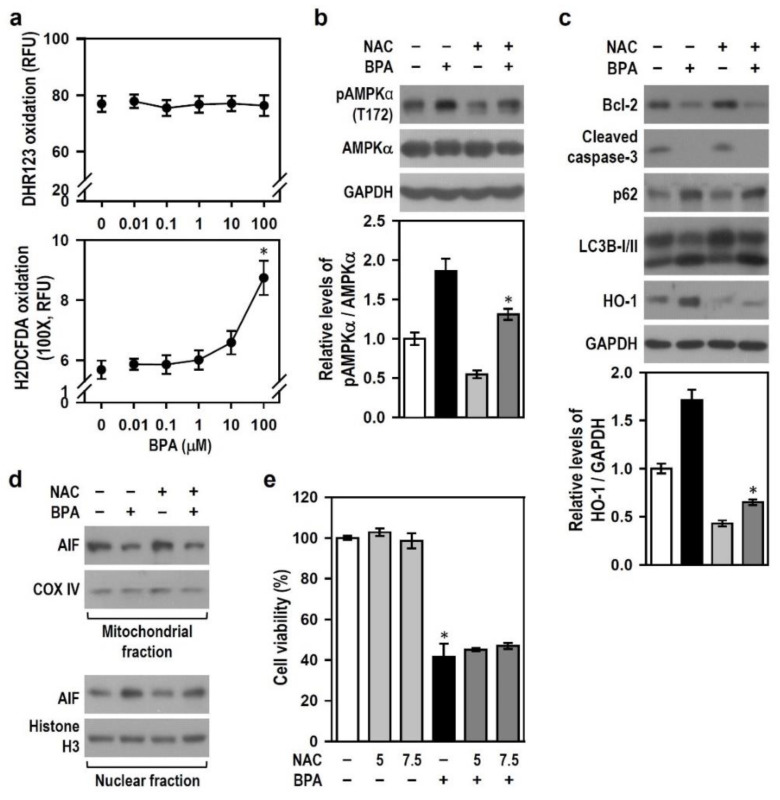
N-acetyl-L-cysteine (NAC) reduces BPA-induced AMPKα phosphorylation and HO-1 production but exerts no effect on stress signalings related to cell degradation. (**a**) Cultures were treated with various BPA concentrations for 24 h and then subjected to the determination of intracellular peroxides and ROS. The line plots show the quantitative data (RFU: relative fluorescence unit), and the untreated cultures (0 μM BPA) served as a control. Each point represents the mean ± SEM (*n* = 4, triplicate). * *p* < 0.05 vs. control group. (**b**) Cultures were exposed or not to 5 mM NAC for 30 min and then treated with or without 100 μM BPA for 24 h. Both pAMPKα and AMPKα levels were detected by Western blots. A bar plot shows the quantitative pAMPKα/AMPKα levels relative to the untreated control group (assigned a value of 1). Data are represented as mean ± SEM (*n* = 4). * *p* < 0.05 vs. BPA alone. (**c**) Similar manipulation was performed as (**b**), and cultures were subjected to Western blots to detect HO-1, Bcl-2, cleaved caspase-3, p62, and LC3B levels. The relative quantitation values of HO-1 level are represented as mean ± SEM (*n* = 4) shown below the representative blot. *p < 0.05 vs. BPA alone. (**d**) Similar manipulation was executed as (**b**). Cultures were harvested and subjected to subcellular fractionation after 12-h incubation. AIF level was detected by Western blots. COX IV and histone H3 were markers standing separately for mitochondrial and nuclear fractions. (**e**) Cultures pretreated with NAC (5 or 7.5 mM) for 30 min were incubated with or without 100 μM BPA for 24 h. A bar plot shows the cell viability percent relative to untreated control group (assigned a value of 100%). Data are represented as mean ± SEM (*n* = 4, triplicate). * *p* < 0.05 vs. control group.

Brain dysfunction caused by BPA is closely associated with oxidative stress, and BPA exhibits potent ROS activity in a variety of tissues to play its toxic role [35,36]. Thus, we examined whether ROS mediates the toxic effects of BPA on N2a neurons. BPA treatment at 100 μM significantly increased the oxidation level of H2DCFDA but not that of DHR123, indicating that BPA stimulates the production of non-peroxide-based intracellular ROS (Figure 9a). To clarify the role of the produced ROS in BPA-induced N2a neurotoxicity, we used the antioxidant N-acetyl-L-cysteine (NAC) to scavenge ROS and combat oxidative stress.

Compared with BPA treatment alone, NAC evidently reduced the level of BPA-induced AMPKα phosphorylation (Figure 9b); this inhibitory effect of NAC could also be observed in BPA-induced HO-1 production (Figure 9c), which indicates that BPA-induced ROS production involves AMPK activation and HO-1 induction. No difference in the production levels of Bcl-2, cleaved caspase-3, LC3B-II, p62, and AIF was noted between the two groups of BPA alone and NAC plus BPA (Figure 9c,d), suggesting that ROS is not associated with BPA-induced apoptosis and autophagy flux inhibition and, thus, may not mediate neuronal death. As expected, NAC was incapable of rescuing cells from the toxic insults of BPA (Figure 9e); this finding clearly indicates that ROS is not involved in BPA-induced N2a cell death. These results suggest that BPA-induced ROS is not responsible for cell death-related apoptosis and autophagy flux inhibition in N2a neurons; instead, the ROS produced may mediate a non-lethal BPA effects involving AMPK activation and HO-1 induction.

## 3. Discussion

BPA is an environmental toxin that has been extensively studied on account of its prevalence in daily life and many human fluids (e.g., urine and blood) and tissues (e.g., kidney, testis, and liver). The detectable distribution of this substance in the brain and cerebrospinal fluid indicates that the blood–brain barrier does not limit the access of lipophilic BPA to the brain. Thus, concern regarding whether BPA exposure causes brain health problems in humans has grown. Reliable evidence supports the supposition that BPA impairs brain development (e.g., neural stem cell proliferation and differentiation) and induces neurodegeneration [2,5,6,37]. Herein, we demonstrated that BPA is directly toxic to differentiating N2a neurons, leading to insults including AIF-dependent apoptosis and impaired autophagy flux. The underlying mechanism involves HO-1 induction and AMPK activation, and the regulatory interplay of both mediates the stress signaling of molecules, including Bcl-2, caspase-3, LC3B-II, and p62, thus leading to N2a neuronal degeneration. The observations agree with in vivo studies by Tiwari et al., which showed that oral BPA administration in animal subjects induced apoptosis and neurodegeneration in the hippocampus region of the rat brain and associated learning/memory deficits [38,39]. Thus, our outcomes may offer a new perspective for the therapeutic strategy against BPA-related neuropathy.

In this study, BPA down-regulated anti-apoptotic Bcl-2 and lowered active caspase-3 to level below the baseline instead of increasing it. Upon an apoptotic insult, mitochondrial AIF undergoes proteolysis and translocates to the nucleus, where it triggers chromatin condensation and large-scale DNA degradation to execute caspase-independent cell death [40]. Thus, we challenged the role of AIF in response to BPA and identified that BPA triggers the mitochondria-to-nucleus translocation of AIF. Because no defects occurred in the pro-apoptotic signals of cytochrome c and caspase-9 prior to caspase-3 cleavage, BPA-induced apoptosis ought to be AIF-driven and caspase-3-independent. As expected, AIF knockdown instead of Z-VAD-FMK administration mitigated BPA-induced survival decline in N2a cells. Our findings contrast the studies suggesting caspase-3-mediated BPA damage [32,41] and are akin to those proposing that AIF-dependent apoptosis is a lethal pathway of BPA cytotoxicity [35]. Moreover, BPA elevated the levels of LC3B-II and p62 with an efficacy similar to that of CQ, an autophagy inhibitor that blocks autophagic flux by decreasing autophagosome–lysosome fusion [42]. This clearly indicates that BPA induces autophagic flux inhibition. Because autophagy is a highly dynamic pathway, the turnover of the related proteins, such as LC3-II and p62, must be artificially blocked to accurately quantify the amplitude of autophagic flux. In particular, p62 involves the formation of ubiquitin-positive inclusions and binds LC3-II to facilitate autophagic degradation [43]. Autophagy activation reduces the generation of p62, while increases in p62 levels infer disrupted autophagic flux because p62 acts as an autophagic substrate and serves as a reporter of autophagic activity [30]. Thus, we recognized that BPA toxicity in N2a neurons includes AIF-dependent apoptosis and impaired autophagy flux. In addition, compared with that treated with BPA alone, an enhanced N2a neurotoxicity observed in the BPA plus CQ treatment may mean that autophagic cell death (type II programmed cell death) is initiated once the significance of autophagy flux inhibition reaches a certain threshold. Further work is needed to investigate this finding.

In response to various forms of stress, cells activate highly conserved heat shock responses by inducing a set of HSPs, which play important roles in cellular adaptation to adverse circumstances [31]. Hence, we inspected the generation of HSPs and verified that BPA only induces HO-1 (HSP32) but not HSP70 or HSP90; moreover, inhibition of HO-1 by SnPP greatly reduces N2a neuronal death. This implies that BPA selectively chooses HO-1 as the mediator responsible for most of its effects on neuronal degeneration. Indeed, BPA-induced HO-1 was positively correlated with the activation of AMPK; down-regulation of Bcl-2, p62, AMPK, and active caspase-3; nuclear translocation of AIF; and increased formation of LC3B-II, all of which led to N2a neurotoxicity. Notably, HO-1 serves as an enhancer to aggravate BPA insults, as evidenced by the observation that hemin aggrades BPA effect on all of these molecules, except p62, and enhances BPA-induced cell death. Hemin has been indicated to be an autophagy activator that elevates autophagic flux and mediates the toxicity via apoptosis, autophagic death, ROS level enhancement, protein modification by oxidized lipids, and mitochondrial dysfunction [44,45]. Thus, in our opinion, hemin cancels p62 accumulation-related autophagy defects and increases the formation of LC3B-II-associated autophagic vesicles in the presence of BPA, which activates autophagic death and leads to greater cell damage than BPA treatment alone.

The role played by HO-1 is highly complex, and the conditions for it to promote neuronal cell survival or induce neurodegeneration are incompletely understood so far [18]. Although studies using animal or cell models have proved the protective efficacy of HO-1, such as antioxidant, anti-apoptotic, and anti-inflammatory activity, based on the observed SnPP neuroprotection against BPA toxicity in N2a cells, we propose that BPA-induced production of HO-1 prompts neuronal degeneration. Actually, up-regulated HO-1 has been widely associated with neuronal damage and degeneration. For example, HO-1 was overexpressed in AD patients’ brains, mainly in the hippocampus and cerebral cortex, and co-localized to neurons, GFAP-positive astrocytes, choroid plexus epithelial cells, ependyma, corpora amylacea, neurofibrillary tangles, and senile plaques [46,47]. HO-1 was strongly expressed in nigral astroglia and dopaminergic neuronal Lewy bodies in Parkinson’s disease [48]. HO-1 up-regulation has been postulated to promote bioenergetics failure, pro-toxin bioactivation, dysfunctional autophagy, and corpora amylacea formation by affecting iron metabolism and mitochondrial activity [49]. Transferrin receptor-independent accumulation of iron and deficits in mitochondrial electron transport may be promoted by enhanced HO-1 activity [50,51]. In fact, HO-1 induction due to genetic manipulation or pharmacological treatments is likely to produce a strong imbalance in the entire HO-1-dependent molecular pathway. For example, deregulation of the balance between HO-1 activity and iron quenching activity results in iron-dependent toxicity. A correlation between HO-1 levels and cytoprotective or cytotoxic outcomes has been noted and points out a threshold for HO-1 up-regulation beyond which the generation of free iron becomes toxic [52].

Earlier studies describing the signaling pathways to up-regulate HO-1 revealed that the Nrf2-dependent activation of HO-1 is generally linked to the protective adaptation of neurons and glial cells, while Nrf2-independent HO-1 activation, which often involves AP-1 or NF-κB, seems to exert neurotoxic effects. For example, in the MPP+-induced parkinsonian rat model, Nrf2 down-regulation and HO-1 up-regulation were associated with the loss of nigral dopaminergic neurons [53]. Brain astrocytes exposed to high glucose underwent apoptosis through HO-1 induction driven by NF-κB and AP-1 [54]; by contrast, Nrf2-dependent activation of HO-1 induced by gintonin or gastrodin protected neurons from neurodegeneration [55,56]. This may be ascribed to the ability of Nrf2 to also activate the gene transcription of other antioxidant and protective proteins involved in iron quenching, chelation, and transport. Given this perspective, examining the upstream signaling of HO-1 induced by BPA is of great interest. More studies on this matter are needed in the future.

We found that, although BPA evokes both acute and chronic AMPK activation/phosphorylation, only chronic AMPK, whose activation time-course is synchronized to the up- or down-regulation of other proteins, was related to stress signaling. By using AICAR or dorsomorphin alone, we determined that AMPK activation and inhibition both trigger apoptosis, as evidenced by the declines in Bcl-2 level and increases in caspase-3 cleavage; of note, the episode of increases in active caspase-3 is repressed by BPA. This indicates that BPA could impair caspase-dependent apoptosis by targeting the formation of active caspase-3 instead of those of cytochrome c and caspase-9. Whilst AICAR prompted AMPK activation and p62 production, it initiated no autophagy and even compromised BPA-induced activation in AMPK and increase in LC3B-II. Hence, we speculate that AMPK activation relieves autophagy and serves as a negative feedback loop that limits the activation/phosphorylation of the kinase itself. Additionally, despite its role as an AMPK activator expected to initiate autophagy, AICAR has been reported to inhibit autophagy in several cell types; this effect is not mediated via its ability to stimulate AMPK but via its inhibition of phosphatidylinositol 3-kinase class III, a kinase essential for autophagy [27]. Thus, in this study, AICAR lessened BPA-induced LC3B-II formation, thereby partially alleviating the autophagy flux inhibition that occurred in BPA-treated N2a neurons. Despite suppressing BPA-induced HO-1 production, AICAR had no effect against BPA insults, which implies that AMPK participates in BPA-induced N2a neurotoxicity but is not as active as HO-1 in mediating toxic BPA effects.

Our data reveal that dorsomorphin alone down-regulates Bcl-2 but up-regulates active caspase-3 and LC3B-II to cause N2a cell damage. This illustrates that AMPK inhibition renders neurons vulnerable to apoptotic and autophagic death. Thereof, AMPK activity is essential to support anti-apoptosis- or anti-autophagy-related cell viability. Indeed, compared with BPA alone, dorsomorphin’s inhibition of BPA-activated AMPK resulted in a severe decrease in N2a cell viability. Considering these findings, we suppose that AMPK activated by BPA is involved in maintaining neuronal survival but scarcely mediates further protection against BPA insults. Suppression of AMPK not only expanded BPA-activated autophagy but also abolished the BPA effects on autophagy flux inhibition, demonstrated by the enhanced LC3B-II formation and reduced p62 accumulation in the dorsomorphin plus BPA treatment. Unlike the results of Vucicevic et al., who found that dorsomorphin (compound C) induces protective autophagy [57], our findings agree with several studies showing that dorsomorphin is a potent cytotoxic agent that kills cancer cells or inhibits glioma proliferation through induction of apoptotic and autophagic death [58,59]. Depending on the conditions such as dose or duration, dorsomorphin may cue cells to undergo either protective or destructive autophagy. Together, in our view, similar to the efficacy of AICAR on p62 up-regulation, AMPK activated by BPA is involved in elevated production of p62 and subsequent p62 accumulation-related autophagy defects; these effects can be erased by dorsomorphin, which initiates AMPK-independent autophagic death to aggravate BPA neurotoxicity.

The partnership or even interactive loop between HO-1 or AMPK is still not fully understood. Reports show that HO-1 up-regulation resulting from pharmacological induction or genetic overexpression enhances AMPK phosphorylation, increases the expression of Beclin-1 and LC3-II and, thus, activates autophagy [22,23]. AMPK activation by AICAR has been indicated to increase HO-1 expression in human smooth muscle and vascular endothelial cells (ECs); HO-1 induction has also been observed in the carotid artery after in vivo AICAR administration [60]. AICAR did not induce HO-1; instead, it reduced HO-1 to levels slightly less than the baseline in porcine aortic ECs [61]. A recent study revealed that AICAR reverses cisplatin-induced increases in HO-1 and declines in phospho-AMPK in NRK-52E rat kidney cells [62]. Disparate results between these studies may be ascribed to differences in animal species, cell types, culture conditions, and/or AICAR exposure times. Nonetheless, in this study, experiments executed using AICAR in combination with or without BPA support the regulatory interplay between HO-1 and AMPK. We determined that HO-1 induction by BPA along with the resultant increase or decrease in signaling molecules, such as LC3B-II and p62, is reversely regulated by BPA-activated AMPK, suggesting an interaction of HO-1 and AMPK involving the antagonistic modulation of common downstream targets. However, alike modulation was not found in Bcl-2 production level; instead, both HO-1 and AMPK caused Bcl-2 down-regulation, perhaps implying Bcl-2 level decline as the common prerequisite for HO-1 and AMPK to mediate the BPA effects on apoptosis and autophagy defects.

Oxidative stress caused by BPA is frequently considered the mechanism underlying BPA-induced cell insults. Thus, ROS scavengers including NAC have been applied to combat BPA toxicity in various tissues/cells. In mouse non-parenchymal hepatocytes, pancreatic INS-1 cells and murine macrophages, pre-incubation with NAC inhibited BPA-induced apoptosis and DNA strand-breaks [35,63,64]. Co-administration of NAC resulted in significant improvements in memory and decreased oxidative stress in BPA-treated rats [36]. BPA inhibited hippocampal neural stem cell proliferation and differentiation and induced apoptosis by increasing oxidative stress; all of which could be mitigated by NAC [37]. Our results agree with other studies in that BPA induces ROS production. However, whilst NAC attenuated AMPK activation and HO-1 induction, it failed to rescue N2a cells from BPA toxicity. The lethal effect of BPA in this study could not be ascribed to oxidative stress, and HO-1 and AMPK activated by BPA-induced ROS may engage with the signalings involving non-lethal episodes. Additionally, NAC did not alter the impacts of BPA on signaling molecules (e.g., Bcl-2, active caspase-3, AIF, LC3B, and p62) related to apoptosis and autophagy defect, accounting for why NAC cannot prevent N2a neurons from BPA-induced survival decline. A recent study, partly similar to our findings, indicated that NAC pre-treatment does not fully inhibit ROS production, leading to non-significant modulation of autophagy marker proteins, such as p62, LC3A/B, and Beclin-1, in primary rat hepatocytes exposed to BPA [65]. BPA cytotoxicity in human promyelocytic leukemia, oral squamous cell carcinoma, and submandibular gland cell lines was not reduced by NAC [41]. Therefore, we are of the opinion that ROS may not be a key factor impacting BPA-induced cell/tissue insults and pathogenetic lesions.

**Figure 10:**
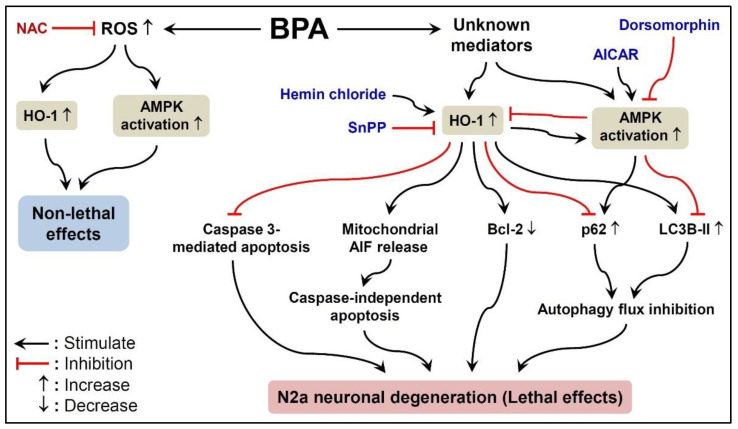
The schematic diagram depicts the degeneration of N2a neurons induced by BPA. BPA, through AMPK activation and HO-1 induction, causes Bcl-2 down-regulation, autophagy defects (characterized by p62 accumulation and LC3B aggregation), and AIF-driven apoptosis, leading to N2a neurotoxicity (e.g., the decline of cell viability and the collapse of neuron-like axon outgrowth). However, caspase-3-mediated apoptosis is not involved.

Our findings reveal the direct cytotoxicity of BPA to N2a neurons. We demonstrate that BPA shows its neurotoxicity by concentration-dependently reducing cell viability and time-dependently down-regulating the neurodifferentiation-related proteins MAP2 and GAP43. BPA causes HO-1 induction and AMPK activation, which, in turn, abolishes caspase-3-driven apoptosis, activates AIF-dependent apoptosis and p62 accumulation-related autophagy defects, and down-regulates Bcl-2; these events cumulatively lead to N2a neuronal degeneration. BPA-induced ROS participates in HO-1 induction and AMPK activation but has no lethal effect on N2a neurons (Figure 10). Thus, HO-1 and AMPK are possible targets for mitigating BPA-induced neurotoxicity; this provides meaningful references for the development of neurotherapies against BPA-related brain degeneration and cognitive deficits.

## 4. Materials and Methods

### 4.1. Chemicals, Antibodies, Kits and Reagents

All chemicals were obtained from Sigma-Aldrich (Saint Louis, MO, USA) if not stated otherwise. Cell culture reagents, dihydrorhodamine 123 (DHR-123) and 2′,7′-dichlorodihydrofluorescein diacetate (H2DCFDA) were purchased from Thermo Fisher Scientific (Waltham, MA, USA). Antibodies, including AIF, phospho-AMPKα Thr172 (pAMPKα T172), AMPKα, HSP70, HSP90, GAP43, MAP2, LC3B, caspase-3, cleaved caspase-3, caspase-9, cleaved caspase-9, and COX IV were purchased from Cell Signaling Technology (Beverly, MA, USA). HO-1 antibody was purchased from Enzo Life Sciences (Farmingdale, NY, USA). SQSTM1/p62, histone H3, Bcl-2, AIF, and cytochrome c antibodies were obtained from GeneTex (Irvine, CA, USA). Caspase-3 and GAPDH antibodies and siRNA were purchased from Santa Cruz Biotechnology (Santa Cruz, CA, USA). Alexa Fluor 488 or 568-conjugated and horseradish peroxidase-conjugated secondary antibodies were obtained separately from Life Technologies (Carlsbad, CA, USA) and Jackson ImmunoResearch (West Grove, PA, USA). The kits used for TUNEL and subcellular fractionation were purchased from BioVision Inc (Mountain View, CA, USA). Pharmacological agents, including SnPP, hemin, AICAR, dorsomorphin, CQ, 3-MA, Z-VAD-FMK, and SIN-1 were purchased from Cayman Chemicals (Ann Arbor, MI, USA).

### 4.2. Cell Culture and Induction of Neuronal Differentiation

Mouse neuroblastoma N2a cell lines, obtained from American Type Culture Collection (Manassas, VA, USA), were grown as monolayer cultures using growth medium containing Dulbecco’s modified Eagle’s medium (DMEM), 10% heat-inactivated fetal bovine serum (FBS), 2 mM L-glutamine, 1% non-essential amino acids (NEAA, 100×), and 1% 100 units/mL penicillin/10 mg/mL streptomycin (100×) and incubated at 37 °C in a humidified atmosphere of 95% air/5% CO_2_. Cells were routinely subcultured in fresh medium after reaching 80–90% confluence every 3–4 days. To induce dopamine neuron-like differentiation, cells were placed in fresh growth medium and incubated for 24 h to allow attachment and recovery. Next, growth medium was replaced with serum-free DMEM supplemented with 1% penicillin/streptomycin, 2 mM GlutaMAX, 1% NEAA, and 0.2 mM dibutyryl cyclic adenosine monophosphate (to induce N2a cell differentiation into dopaminergic neurons) in the presence or absence of BPA and/or various chemical agents. The cultures were then re-incubated for the indicated time length.

### 4.3. Quantification of Axon Outgrowth

Neurotoxicity was evaluated by quantifying axon outgrowth levels in differentiating N2a neurons. Cells were seeded in 6-well plates at a density of 2 × 10^4^ cells/well and then incubated in the indicated treatment for axon outgrowth quantification. Cells with axon-like processes, which are defined as cellular extensions greater than two cell-body diameters in length, were scored as differentiated cells under an inverted light microscope at 200× magnification. The number of differentiated cells in a single microscopic field was counted and then calculated as a percentage relative to the total cell number. At least 10 randomly selected fields were examined for each treatment group per experiment.

### 4.4. 3-(4,5-Dimethylthianol-2-yl)-2,5-Diphenyltetrazolium Bromide (MTT) Reduction Assay

Neurotoxicity was assessed by determining N2a cell viability. An MTT reduction assay was used to measure the metabolic activity of living cells and reveal the data proportional to the number of viable cultured cells. MTT (0.5 mg/mL) dissolved in phosphate buffered saline (PBS) was added to the cultures for 2 h of incubation at 37 °C. After removing the medium containing MTT, the purple formazan crystals formed were dissolved with dimethyl sulfoxide. The absorbance of the resulting solution was obtained by measuring at 570 nm using a Multiskan microplate photometer (Thermo Fisher Scientific, Waltham, MA, USA) and subtracting the reading at 620 nm (reference wavelength). Blank values were subtracted from each reading, and the absorbance was expressed as a percentage relative to the corresponding control.

### 4.5. Determination of Intracellular Oxygen Free Radicals

To assess intracellular oxygen free radical level, the fluorescence intensity of the cultures was determined after loading with the fluorogenic probes H2DCFDA, which detects reactive oxygen species (ROS) to indicate the degree of oxidative stress, and DHR-123, which detects peroxynitrite and peroxides. The cultures were washed once and replaced with fresh PBS. H2DCFDA (10 μM) or DHR-123 (20 μM) was then added to the cultures for 30 min at 37 °C. An FLx800 fluorescence microplate reader (BioTek, Winooski, VT, USA) was used to detect the fluorescent signals. The resultant fluorescence of H2DCFDA undergoing ROS oxidation in cultures was monitored at λex/λem = 485/528 nm, while DHR-123 oxidation product fluorescence was measured at λex/λem = 488/515 nm. All fluorescence readings (relative fluorescence unit, RFU) were normalized to the background value (i.e., autofluorescence in cultures without the fluorescent probes) and then compared with those of the untreated control groups.

### 4.6. TUNEL Assay

Apoptosis was assessed by TUNEL assay kits according to the manufacturer’s instructions. TUNEL-positive cells were observed under an inverted fluorescence microscope (Nikon ECLIPSE Ts2 microscope, Nikon, Inc., New York, NY, USA), equipped with a digital camera (DS-Fi3, Nikon, New York, NY, USA). Matching bright-field and fluorescent TUNEL-FITC photos were obtained from at least 30 fields for each treatment group per experiment for cell quantification. The number of cells presenting fluorescent TUNEL-positive nuclei in a single field was calculated as the proportion of the total cell number in the matching bright-field photo.

### 4.7. Immunofluorescence Staining

Cells grown on glass coverslips (22 mm × 22 mm) were fixed with 4% paraformaldehyde, permeabilized with 0.5% Triton X-100 in PBS, blocked with 3% BSA/2% goat serum, and incubated with primary anti-LC3B or AIF antibody at 4 °C overnight. After washing, the cells were incubated with the corresponding secondary antibody conjugated with Alexa Fluor 488 or 568 in the dark for 2 h and analyzed under an Axioplan2 fluorescence microscope (Zeiss, Oberkochen, Germany). Anti-fade mounting medium (Vector Laboratories, Burlingame, CA, USA) containing 4′,6-diamidino-2-phenylindole (DAPI) was applied to air-dried coverslips to visualize nuclei. Autophagy was assessed by observing cells displaying punctate LC3B fluorescence, and the total number of LC3B puncta per DAPI-labelled cell was counted. At least 250 cells were examined for each treatment group per experiment, and the numbers of LC3B puncta formed per cell were statistically compared between treatment groups.

### 4.8. RNAi and Transient Transfection

Prior to transient transfection, cultures were maintained in antibiotic-free medium for 18–24 h. Cells were transfected in serum-free DMEM with Lipofectamine 2000 transfection reagent (3 µg/well; Thermo Fisher Scientific, Waltham, MA, USA) containing 50 nM siRNA according to the manufacturer’s protocol. After 6 h of incubation, the medium was replaced with fresh growth medium containing 10% FBS, and the cells were cultured for an additional 48 h. Western blots were subsequently used to determine the RNAi effect on target proteins. All purchased siRNA oligos were designed and synthesized by Santa Cruz Biotechnology. Mouse AIF-siRNA (sc-29194) consisted of a pool of 4 target-specific 19–25 nt siRNAs. Control-siRNA (sc-44230) was a non-targeting 20–25 nt siRNA designed as a negative control that leads to no degradation of any cellular message.

### 4.9. Subcellular Fractionation

Commercially available kits (K256 and K266) were applied to isolate the cytosolic, nuclear and mitochondrial fractions of the cell according to the manufacturer’s instructions. Protein concentration was determined using the Bio-Rad protein assay (Bio-Rad, Hercules, CA, USA) based on the Bradford dye-binding method.

### 4.10. Western Blots

Cells were harvested in ice-cold RIPA buffer (50 mM Tris/HCl, pH 8.0, 150 mM NaCl, 1% Nonidet P-40, 0.5% sodium deoxycholate, 0.1% SDS) containing a cocktail of protease and phosphatase inhibitors (Sigma-Aldrich). Lysis was performed on ice for 20 min, and the total lysates were centrifuged at 14,000× *g* for 10 min (4 °C). A Bio-Rad protein assay was used for protein quantification. Cell lysates (≥30 μg) were resolved by SDS-PAGE and electroblotted onto polyvinylidene fluoride membranes (Millipore, Bedford, MA, USA). After blocking with 5% non-fat dry skim milk, the membranes were immunoblotted for the desired proteins. GAPDH served as a protein loading control. The immunoblots developed on X-ray film were visualized by an enhanced chemiluminescence system (Pierce; Thermo Fisher Scientific), and the intensity of protein bands was quantified using Image J software (NIH, Bethesda, MD, USA).

### 4.11. Statistical Analysis

SigmaStat version 4.0 software (Jandel Scientific, San Rafael, CA, USA) was used for statistical analysis. Statistically significant differences among multiple treatment groups were analyzed by one-way ANOVA, followed by Dunnett’s test or Bonferroni’s *t*-test. Paired groups were compared using Student’s t-test. Values are expressed as mean ± standard error of the mean (SEM). A value was considered significant if its probability was less than 0.05 (*p* < 0.05).

## Data Availability

The data that support the findings of this study are available from the corresponding author upon reasonable request.

## Supplementary Materials

The supplementary data can be found online at https://www.mdpi.com/article/10.3390/ijms222010948/s1.
