# Supplementary material for: Bisphenol a Induces Autophagy Defects and AIF-Dependent Apoptosis via HO-1 and AMPK to Degenerate N2a Neurons
Source: Int J Mol Sci. 2021 Oct 11;22(20):10948. doi: 10.3390/ijms222010948 (PMC8535739; doi:10.3390/ijms222010948)
Supplement: Supplementary file 1 [file ijms-22-10948-s001.zip › ijms-1384160-supplementary.pdf]

Supplementary Materials

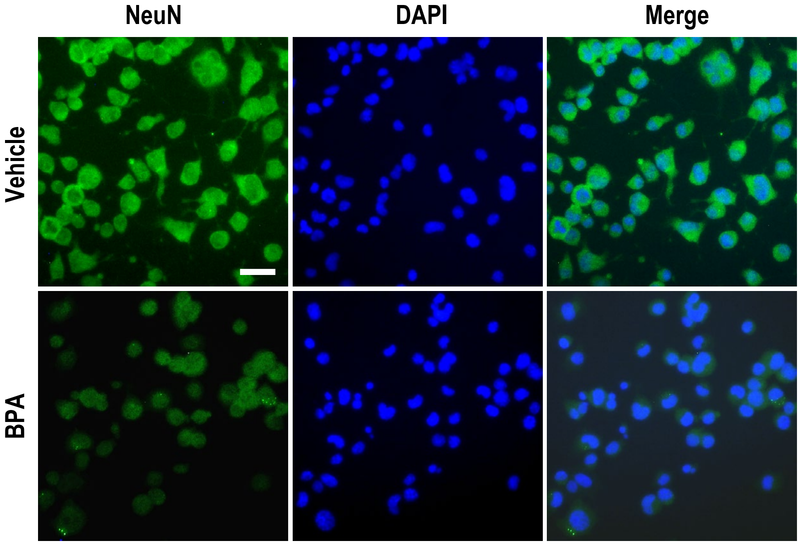

Figure S1. The decrease of neuronal nuclear protein (NeuN) immunoreactivity is detected in BPA-treated N2a cells as examined by fluorescence microscopy. Vehicle- and BPA-treated N2a cells were immunostained with an anti-NeuN antibody (green) and DAPI (blue, cell nuclei labeling) at 24 h. Vehicle-treated culture served as a control. Representative photos were shown to indicate the reduced NeuN protein expression in BPA-treated cultures compared to vehicle-treated cultures. Moreover, several nuclei in BPA-treated N2a cells became irregular, shrunken or smaller as the nuclear density decreased. Scale bar = 50 μm.

Supplementary Materials

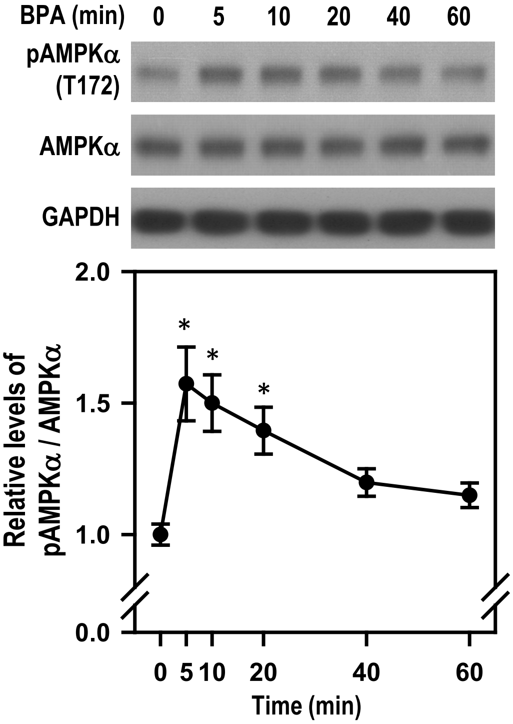

Figure S2. BPA induces a rapid AMPK phosphorylation. Cultures were treated with BPA (100 μM) for 0 to 60 min. The pAMPKα and AMPKα levels were detected by Western blots. The untreated culture (BPA treatment for 0 h) was regarded as the control group. A line plot reveals the quantitative values of pAMPKα (normalized by AMPKα) relative to the control group assigned a value of 1. Each point represents the mean ± SEM (n = 4). *$p < 0.05$ vs control group.
